# Geoelectric field response characteristics analysis of floor roadway surrounding rock fracture caused due to coal seam mining

**DOI:** 10.1038/s41598-021-01823-0

**Published:** 2021-11-16

**Authors:** Yuanchao Ou, Pingsong Zhang, Maoru Fu, Xiongwu Hu, Rongxin Wu, Chang Liu, Binyang Sun, Shiang Xu, Shenglin Li, Lei Tan

**Affiliations:** 1 State Key Laboratory of Mining Response and Disaster Prevention and Control in Deep Coal Mines, Anhui University of Science and Technology, Huainan, 232001 Anhui China; 2 School of Earth and Environment, Anhui University of Science and Technology, Huainan, 232001 Anhui China

**Keywords:** Natural hazards, Solid Earth sciences, Energy science and technology, Engineering, Physics

## Abstract

The fracture of rocks surrounding the floor roadway during the mining of the working face of a coal mine is a complicated spatiotemporal process due to the superimposed action of multiple stress fields on the surrounding rock mass. Using the surrounding rock of a floor roadway in the working face of the Huainan Pan’er Mine as the research subject, we conducted real-time monitoring using geoelectric field monitoring technology, and found the spatiotemporal response law of the geoelectric field in the process of regional rupture and damage of engineering rock masses under a complex stress field environment. The results show that (1) the time series response characteristics and spatial distribution of the geoelectric field signal are closely related to the stress distribution and damage evolution of the surrounding rock mass; (2) the rupture and damage degree of the goaf floor significantly increased when the working face was pushed through the monitoring area for 20–40 m. During this process, the excitation current dropped by 4–12 mA, and the self-potential pulse fluctuation amplitude was greater than 400 mV; (3) from the beginning of the monitoring process to the end of the monitoring, the self-potential in the damaged area decreased by 250 mV, and the self-potential in the mudstone layer below the damaged area increased by 140 mV. The electrons released into the environment around the damaged rock mass during the severe impact phase of mining did not flow back to the damaged area, and the positive charge in the damaged rock mass gradually accumulated in the complete rock mass in units of rock strata; (4) when superimposed and supported by anchor rod and cables, the bearing capacity of the shallow bearing circle of the roadway was enhanced, and the excitation current presented a step-like overall increase during mining of the working face with a small drop after every significant increase. This result is of significance in monitoring the evolutionary process of real-time failure of rock masses under complex stress environments using geoelectric field information and in improving the quality of geoelectric field monitoring technology testing applications in the future.

## Introduction

Numerous types of intelligent mechanical mining equipment have been successfully developed and are currently used, and they significantly improve the efficiency of coal seam mining^1–3^; however, roadway construction in mines requires a considerable amount of time. It is imperative to change the problems of single-use and short service life of previous roadways and design roadways that can be reused and remain safe and stable during multiple mining of the surrounding working faces. Thus, research on the stability and failure of roadway-surrounding rock during coal seam mining is of great significance to develop a technical framework for roadway-surrounding rock stability control and engineering.

Over the years, problems with the mechanism, maintenance, deformation control and failure of roadway surrounding rock^4–8^ have been the focus of academic research and engineering construction. Many studies have tested the deformation and fracture of roadway surrounding rock, including sonic wave testing^9^, stress and strain test^10^, multipoint displacement test^11^, borehole camera technology^12^, and resistivity test^13^.

The borehole electrical method uses the resistivity value as a parameter to evaluate and analyse the risk of rock deformation and failure or water inrush from the working face^14–18^. However, this parameter is unstable, has low accuracy and high interference, and often leads to large jumps and fluctuations in the resistivity value, which impact the final result. Geoelectric field monitoring based on the borehole electrical method has many advantages, such as high adaptability, low interference due to abnormalities, robust test results, simple data processing, and high reliability of test data. In recent years, there has been increasing scientific research on its technical theory and applications^19–21^. For example, Liu et al. designed three types of seepage model tests and studied the correlation between the excitation current and the water inflow of the test model; their study revealed the space–time evolution of groundwater seepage systems in the process of mining and the geoelectric field response characteristics of mine water inrush disasters^22^. Wu et al. revealed the change law of the spontaneous potential in the process of rock mass damage based on numerical simulation, physical simulation test and field monitoring and further mastered the self-potential response characteristics of "three zones" of a coal seam roof in the mining process^23^. Wu et al. expounded on the principle of the electrode current method and proposed the method and construction process to monitor mining overburden damage using the borehole electrode current method, which achieved a good application effect in field measurements^24^. Liu et al. provided a detailed description of the point discharge occurrence, and the micromechanism of the self-potential anomalies was explained. Combined with laboratory and in situ test results, it is proven that the spontaneous potential of a rock mass impulsively fluctuates and decreases as a whole in the process of continuous damage^25^. Yang et al. studied the coupling relationships between stress, self-potential, and acoustic emission parameters for bituminous and anthracitic coals under uniaxial compression^26^.

In terms of underground practical applications, ordinary strength cables are mainly used in monitoring holes. This type of cable has low tensile strength and no steel wire or other protective components inside. When the rock mass is greatly deformed or broken, the cable will be damaged, so it cannot continue to collect later data. In addition, the rock mass damage and failure of the roof and floor of the working face are due to the change in the rock mass stress field caused by mining. The stress field environment is relatively single and does not involve rock mass failure under the superposition of multiple complex stress fields. The analysis of geoelectric field monitoring data is relatively simple. Meanwhile, the underground in situ monitoring of geoelectric fields in the existing literature mainly selects excitation currents or self-potentials for testing, while there are few engineering research results for a comprehensive comparative analysis of these two electrical datasets.

In this regard, the author takes the floor roadway of a working face in the Huainan Pan’er mine as the engineering background. By constructing the monitoring hole in the floor roadway and using the reinforced cable, the survival condition of the sensor in the hole under the complex stress environment is obviously improved. The rock mass damage process in the monitoring area during the entire mining cycle of the working face is effectively obtained. To master the fracture evolution characteristics of floor roadway surrounding rock under a complex stress environment, electrical parameters such as natural potential and excitation current are monitored in real time. This paper reveals the generation mechanism and variation characteristics of electrical anomalies in the process of rock mass damage, analyses the relationship between self-potential and excitation current, and discusses the advantages and application effects of geoelectric field tests in a complex test environment affected by the superposition of multiple stress fields. The research results lay a foundation for further comprehensive utilization of the geoelectric field technology to accurately identify the rock mass damage and failure and data fusion processing in complex environments.

## Geoelectric field monitoring technology

### Technical principle

**Figure 1:**
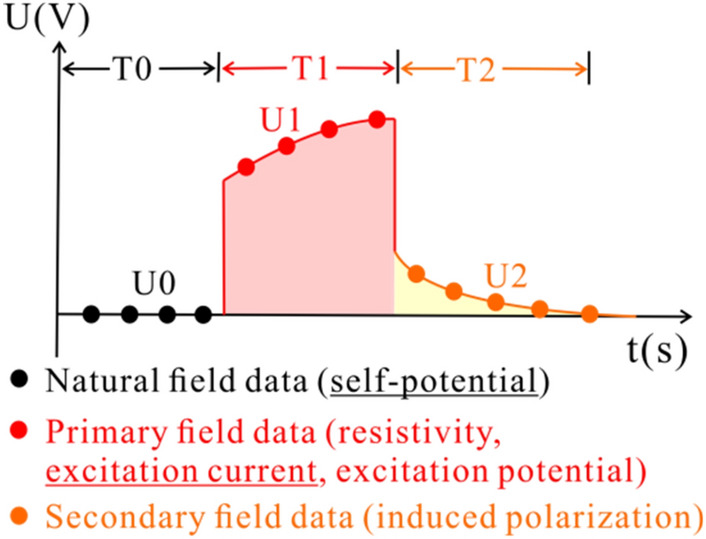
Potential time series of a single electrode.

When the internal stress of the rock mass in the deep environment of the mine accumulates or releases to a certain extent, it will lead to the fracture damage of the rock mass. In the process of rock mass fracture, it will be accompanied by micro or macro abnormal manifestations such as electromagnetic, acoustic wave and temperature. The occurrence of these physical phenomena will inevitably lead to the information mutation of the physical field and chemical field. By monitoring the geoelectric field in the rock mass, the electrical data of three stages: natural field, primary field and secondary field can be observed (Fig. 1).

The existing research results have confirmed that there is charge separation at the crack tip in the process of rock failure. The highly concentrated stress causes the energy release of rock. The escape of charged particles, as a form of energy release, will lead to abnormal self-potential of the tested rock. Self-potential mainly shows a sudden pulse like continuous fluctuation and a slow decline as a whole. For the primary field, the overall conductivity of the fracture developed area in the rock mass becomes weak, and the current line in this area is repulsed. The current decreases during fracture development. Therefore, when the power supply voltage is fixed, the damage state of rock mass can be inferred according to the change of excitation current^22,25,27,28^.

### Field applicability analysis of the test technology

**Figure 2:**
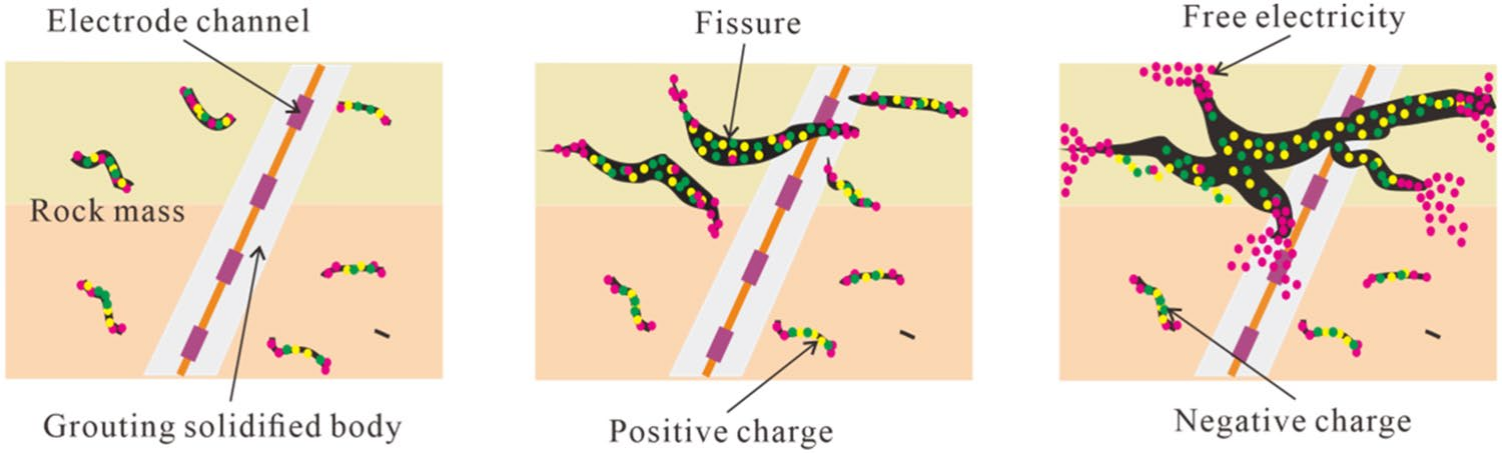
Schematic diagram of the monitoring of the geoelectric field of the rock mass fracture^25^.

Mining of the working face changes the original stress distribution in the surrounding rock mass, which causes the movement, cracking, or even fracture of the roof and floor rock mass. The roadway-surrounding rock mass located under the coal seam is subjected to the original rock stress, mining stress, and support stress, which dynamically change when the working face is mined. The change in the stress field in the rock mass and the development of microcracks and macrofractures lead to real-time changes in geoelectric field information^24,25^. By monitoring any change in the geoelectric field signal in the rock mass or any abnormal response characteristics, one can comprehend the evolution process of the real-time failure of the rock mass in different areas in the roadway-surrounding rock and take targeted measures for prevention (Fig. 2).

**Figure 3:**
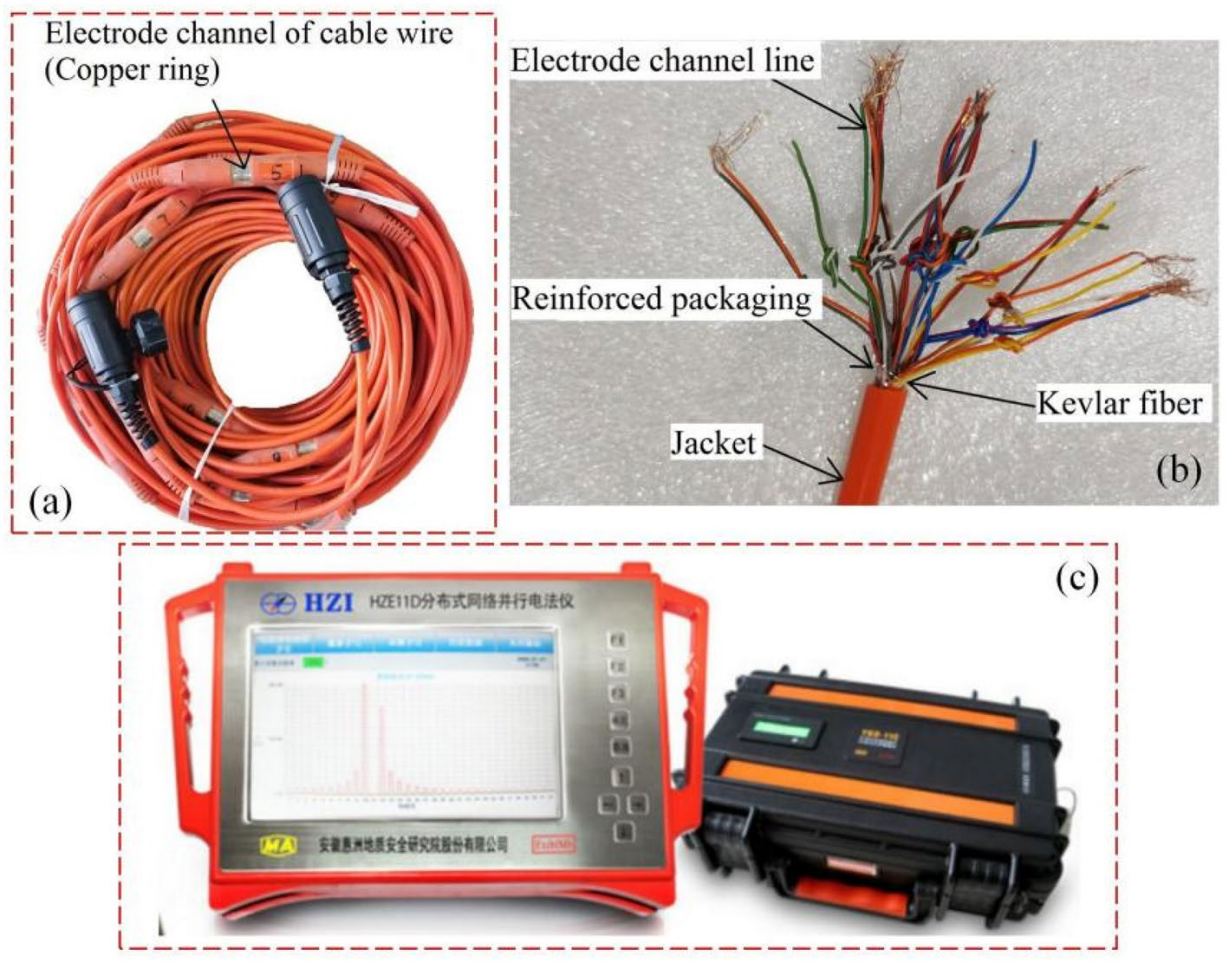
Geoelectric field monitoring equipment. (**a**) Electrical cable, (**b**) electrical cable structure details, (**c**) parallel electrical method.

After drilling monitoring holes into the bottom roadway to the coal seam of the working face, an electrical measurement sensor cable was implanted into the hole, followed by injection of grout until it was well coupled with the cable electrode. After the grout solidified and was as strong as the surrounding rock, the Parallel Electrical Method^28^ (PEM) was used to collect the background geoelectric field information before coal was mined from the working face (Fig. 3). As the working face gradually approached and crossed the monitoring hole, the sensor continuously monitored the changes in natural potential and excitation current in the process of surrounding rock failure in real time under the impact of the dynamic pressure.

It should be noted that monitoring boreholes generally pass through multiple rock formations with different lithologies. The slurry contacted by the sensor was made as close as possible to the mechanical properties of the original rock around the borehole to effectively transmit the rock mass stress. In this study, field drilling grouting adopts the segmented grouting operation mode. The conductivity of different rock strata is different. In the non-mining stage of the working face, the measured background excitation current of the rock strata with good conductivity will be larger; otherwise, it will be smaller. However, the conductivity difference of different rock layers does not affect the analysis and judgement of monitoring results. The focus of research and analysis is to compare the changes before and after each measurement point and determine the damage status of the rock mass in the corresponding measurement point area according to the fluctuation law of the data. Precisely because different rock strata have different physical properties, when bearing the same size of stress, the damage and deformation degree of rock masses in different rock strata is also different, and the geoelectric field information will be clearly reflected.

Furthermore, cracks or pores may appear during slurry solidification, but the pore diameter of the borehole is very small compared with the large-scale surrounding rock. Moreover, the information that the sensor can capture is not limited to the borehole; it is the result of comprehensively reflecting the damage status of the rock mass in a certain area. In addition, there will be different degrees of natural cracks and other defects in different original rocks. Existing studies have proven that there are near-field effects and far-field effects in geoelectric fields, but there is no quantitative understanding of how much range of rock mass fracture information can be reflected by the sensors. These factors will not greatly affect the applicability, feasibility and reliability of geoelectric field monitoring technology in complex geological environments.

The range of the self-potential or excitation current measured by the electrode sensor is mainly constrained by the spatial scale or damage degree of the test object. The objects in this study have a large spatial scale and significant damage changes. The fluctuation range of the self-potential reaches hundreds or even thousands mV, and the excitation current fluctuates in the range of tens of mA. The response of the sensor to the crack is mainly affected by the damage state of the rock mass in terms of the development size of a single crack, number of cracks and distribution density of cracks. Therefore, even if the fracture development is small, if the number is sufficiently large and densely distributed, the geoelectric field will also have an obvious response. In contrast, when there are only a few fractures, even if they are very developed, the geoelectric field information captured by the sensor is not necessarily obvious.

Through the specific discussion of the above problems, it is helpful for readers to better understand the working principle and engineering applicability of the geoelectric field testing technology.

## Engineering monitoring and research

### Engineering geological conditions

**Figure 4:**
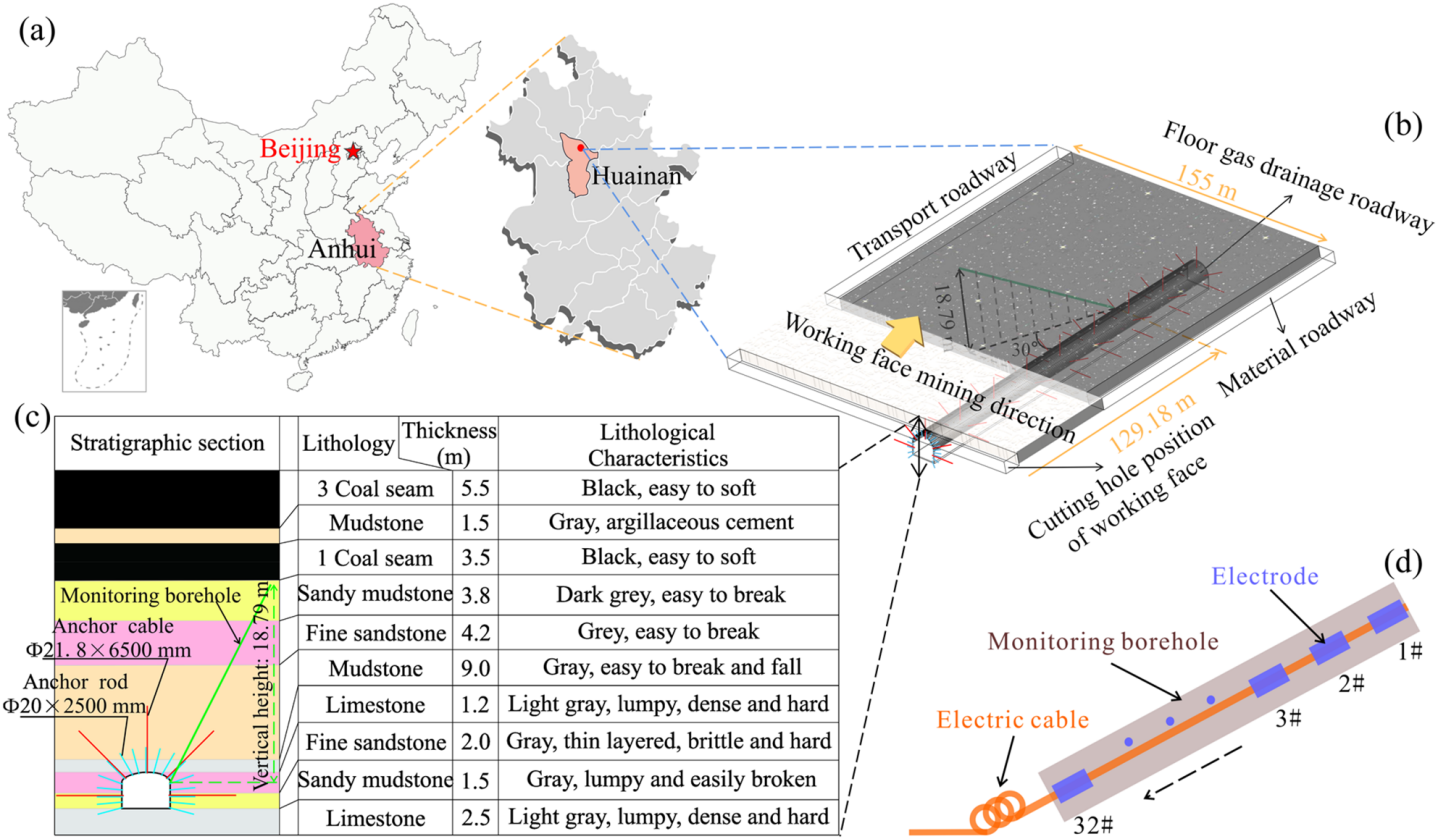
Location of the research area and cross-section of the strata structure.

This project is located in a working face of Pan’er Mine in Huainan. The elevation of the working face was − 450 to − 490 m, the average buried depth was about 470 m, the minable strike length was about 1345 m, and the inclination length was 155 m. The main coal mining was 3#, the average thickness of the coal seam was 5.5 m, and the bulk density was 1.34 t/m^3^. The roof is managed by the method of full caving along the long wall, and the actual mining thickness of the working face was about 5 m. There are no major faults or structures within the working face. The lithology and other related information of the floor of 3# coal seam is shown in Fig. 4.

The floor gas drainage roadway was located in fine sandstone and sandy mudstone, with a width × height of 4600 mm × 3500 mm. The top and bottom rock layers consisted of hard, dense limestone. The roadway was used mainly for the placing and installation of safety equipment and systems for gas drainage and ventilation. The support scheme of the floor gas drainage roadway adopts the support form of “bolt + anchor net + local anchor cable and grouting”. Some fractured or weak surrounding rock sections are strengthened in advance. The anchor is a strong pre-tensioned anchor, the anchor is Φ20 * 2500 mm, the spacing is 700 × 700 mm, the anchor cable is Φ21.8 * 6500 mm, and the spacing is 1900 × 2000 mm. According to the symmetrical distribution, each row has five; the spray layer adopts C20 concrete with a thickness of 100 mm.

### Monitoring hole design

The monitoring orifice was 129.18 m away from the cut-out position of the working face, the borehole points to the cut-out direction of the coal seam in the working face, the actual length of the borehole was about 46.20 m, and the vertical height of the borehole was controlled to be 18.79 m. The cable in the hole was mine used high-strength tensile and compressive cable with electrode spacing of 1.5 m and a total of 32 electrodes. It should be noted that the 32# electrode which should be placed outside the monitoring orifice was also fixed inside the orifice. The specific space layout of monitoring hole relative to working face is shown in Fig. 4.

### On-site data collection

**Figure 5:**
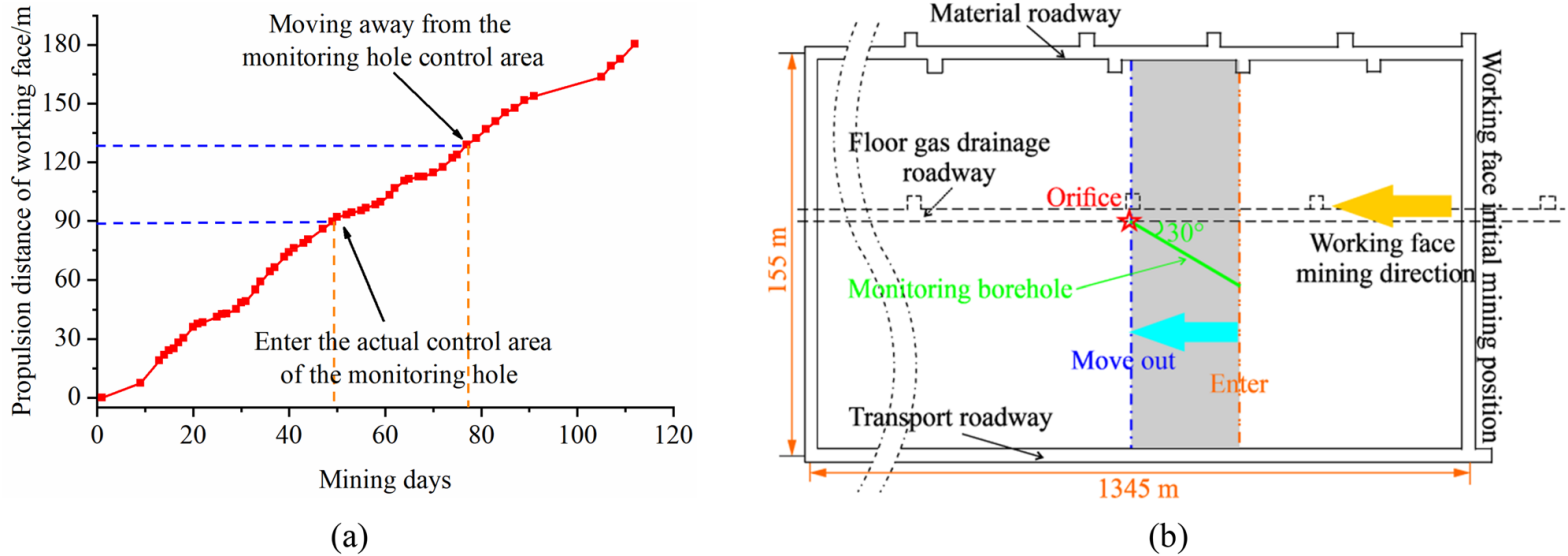
Relationship curve between data collection and mining position of working face.

The field monitoring lasted for 112 days. During this period, 57 times of geoelectric field data were collected by using the Parallel Electrical Method (PEM) instrument, and the whole process of roadway surrounding rock failure evolution in the front, middle and back stages relative to the monitoring position was completely captured. Among them, the acquisition mode of the instrument was single point electric source field testing method (AM), the power supply time was 0.5 s, the sampling time interval was 50 ms, and the power supply mode was single positive method^28,29^. The mining speed of on-site coal seam working face was relatively stable, about 1.6 m/d. The specific data acquisition is shown in Fig. 5. The monitoring hole control area referred to in the figure is only to represent the relationship between the corresponding acquisition time and mining position when the working face is mined to the area directly above the monitoring hole. It should be noted that it is not the monitoring hole sensor that can only capture the data information within this time period.

According to the data provided by the mine during the monitoring period and the observation of the field data collection personnel, it is known that there is no water outflow phenomenon in the roof and floor of the coal seam and the bottom drainage roadway during the whole monitoring period. Considering that the working time of underground coal mining team and industrial electric field may cause interference to the data. To minimize the impact of industrial electric field and other noise. We choose to collect field data at 2 p.m. every day, during which the underground coal mining operation is stopped. To ensure the quality and reliability of field data, the sequence of each electrical data acquisition is: self-potential, excitation current.

## Analysis of in situ test results

### Evolution of geoelectric field monitoring data

**Figure 6:**
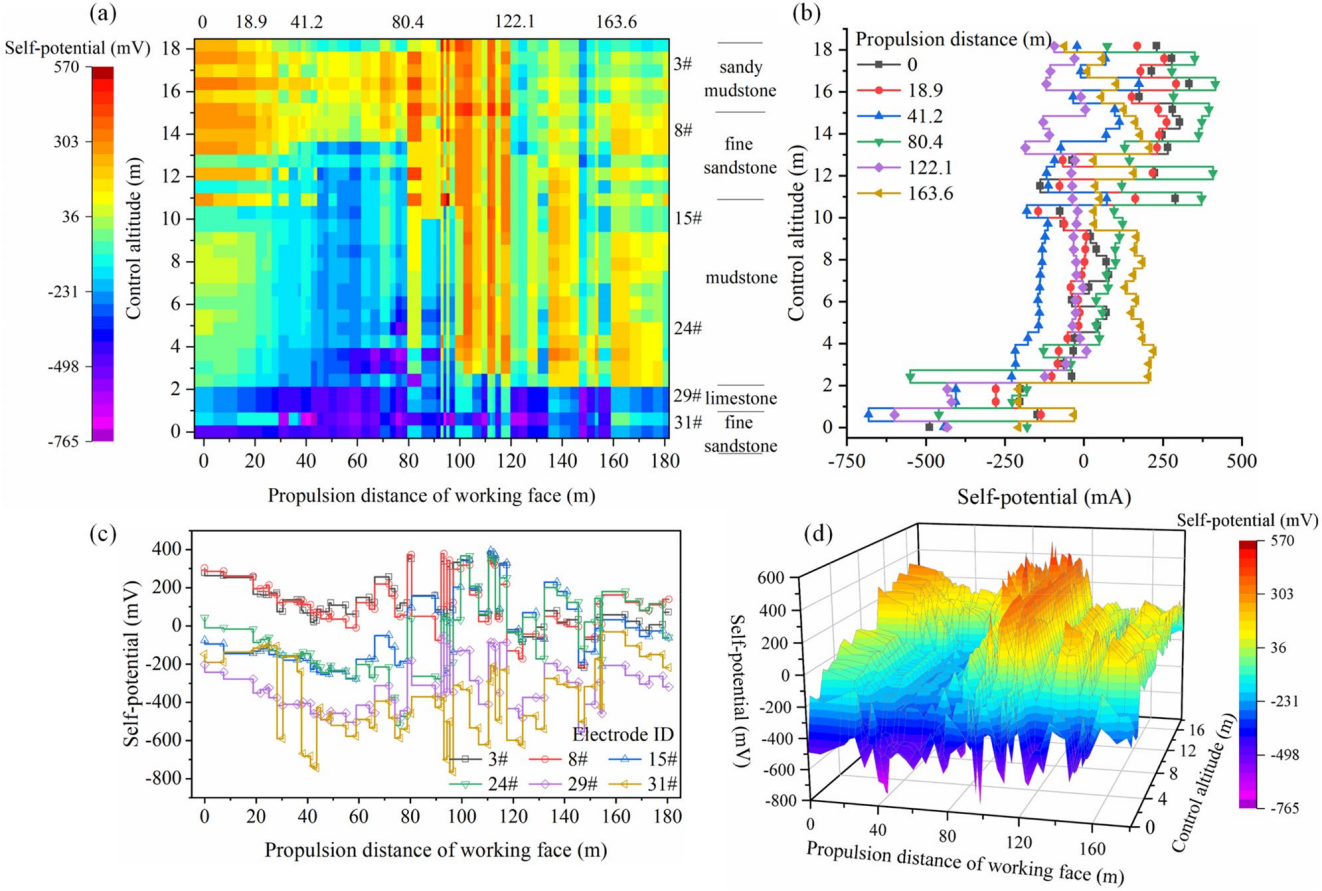
Self-potential changes in the roadway-surrounding rock during mining.

**Figure 7:**
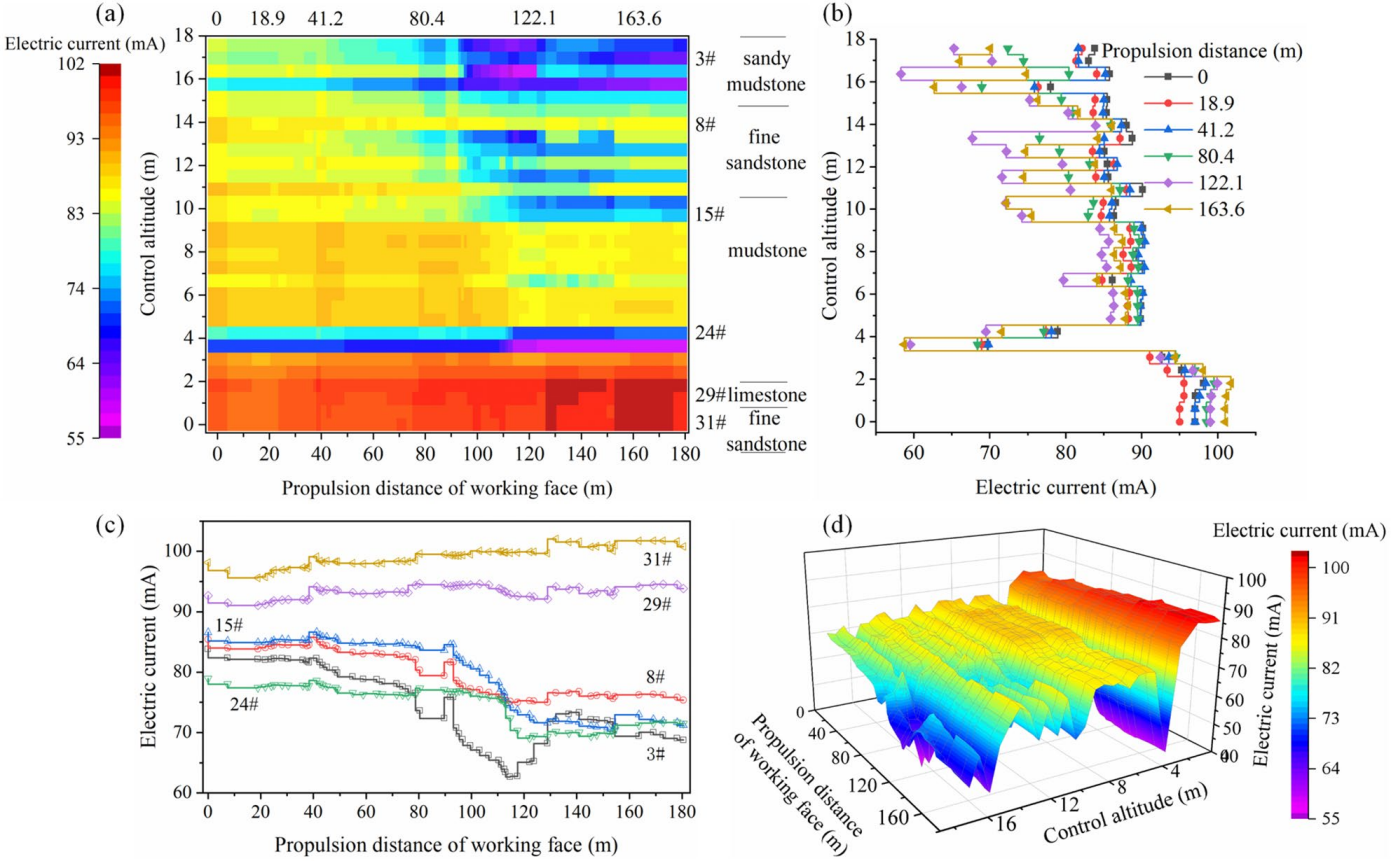
Changes in excitation current in the roadway-surrounding rock during mining.

Due to the superposition of multiple stress fields (the original rock stress, mining stress, and support stress), the response characteristics of the geoelectric field in the rock mass vary in different regions during the monitoring period. The in situ test data results in the graph show the evolution of the geoelectric field due to the rupture of the surrounding rock mass in the test area during coal mining (Figs. 6 and 7).

When the advancement distance of the working face is 0–50 m, the monitored area is far from the mining location, and the surrounding rock mass is less affected by the lead abutment pressure. The rock mass is in the elastic deformation stage, and the pores or original fractures in the rock mass are closed due to compression. The self-potential shows a slow downward trend with a drop of less than 200 mV, and the excitation current does not significantly change, with the fluctuation range within 3 mA.

When the working face advances to 80 m, the lead abutment pressure on the floor increases, and the rock mass in the monitored area undergoes compression with plastic deformation. The microcracks initiate, expand, and connect in the shallow rock mass of the floor, and some macrocracks appear. The downward trend of the self-potential ends. With the development of microcracks, the self-potential shows a small increasing fluctuation, which increases to within 100 mV; the excitation current of electrode #3 shows a significant drop of 5 mA, while the excitation current of electrode #8 more slowly drops. The macrocracks in the floor develop in sandy mudstone and tend to spread down to fine sandstone. In the surrounding rock mass of the roadway supported by bolts and anchor cables, the excitation current presents a characteristic increase in the step fluctuation with an amplitude less than 2 mA, and there is a small decrease before every increase.

As the working face advances, it enters the upper part of the monitored area. Here, the floor rock mass is severely affected by mining. The rock shifts from compression to expansion and enters a state of plastic yield. The macroscopic cracks inside the rock mass rapidly expand and penetrate. A wide range of progressive damage occurs, and the volume expansion is accelerated. In this stage, the self-potential is typically a pulse-like violent fluctuation with a maximum fluctuation amplitude of 500 mV, and the influence range extends to the entire rock mass in the monitored area. The self-potential in the mudstone suddenly shifts from a negative value to a positive value. However, the excitation current does not significantly change, which implies that the mudstone layer is not macroscopically damaged. Due to the severe macroscopic damage of the rock mass at this stage, the emission of enriched free electrons and diffusion of positive charges appear in the fracture zone. The spatial impact of this phenomenon is significantly expanded, and the rock mass in the rock formation remains affected even without macroscopic rupture. As the working face advances through the measuring points, the stress distribution of the floor rock mass in the goaf is adjusted, which results in unloading damage and further aggravates the damage of the floor rock mass, and the excitation current in the rock formations (sand mudstone and fine sandstone layers) significantly decreases within a range of 4–12 mA within the monitoring hole control height range of 9–18 m.

When the working face moves away from the monitored area, the overlying rock in the goaf collapses to the floor. The floor rock is compressed, and stress continues to be transmitted by the upper broken coal rock mass. Before the stress distribution is adjusted, the rupture zone of the floor rocks experiences macroscopic crack compression, closure, and new cracks, but it generally stabilizes. At this point in time, the self-potential fluctuation amplitude in the floor rupture area is weakened, and the positive charge moves and becomes concentrated in the undamaged mudstone layer; the excitation current increases, and there is a more significant increase in the sandy mudstone layer closer to the working face floor than the fine sand layer below it, which shows that the shallow rock mass of the floor is more affected by mining and caving compaction than the deep rock mass during the actual mining process. When the distance of the working face reaches 154 m, the rock mass in the monitored area is in a stable state, and the self-potential of the mudstone layer at this time is higher than that during the initial period. The final monitoring data show that the electrons released from the damaged rock mass do not flow back in a short period.

During this process, the damage and fracture of sandy mudstone and fine sandstone closer to the working face floor are due to the stresses from the original rock and mining. However, the surrounding rock mass in the roadway anchorage zone is affected due to the superposition of the supporting stress with the above two stresses. Thus, the mechanism of rock deformation and damage in different monitoring areas differs, and there is a difference in the response characteristics and evolution of the geoelectric field. In this section, we conduct an in-depth analysis of the floor failure zone and briefly describe the characteristics of the geoelectric field in the roadway support zone. The failure characteristics and evolution mechanism of roadway support areas will be analysed in the next section.

### Fracture process analysis of the roadway surrounding rock zone

After excavation of the roadway, the radial stress on the surrounding rock is released, and the hoop stress increases. Damage occurs in the surrounding rock mass in the form of cracks and the formation of loose rings on the roadway. The use of high-stress superstrong bolts and anchors, bolt mesh, and shotcrete increases the normal constraint so that the deformation of the surrounding rock is slowed down, and hoop stress peaks develop towards the deep part of the roadway-surrounding rock. Using anchor cable support, the radial stress on the surface of the surrounding rock and the hoop stress are transmitted deeper, so the range of the load-bearing ring increases, and the anchor rod, anchor cable, and broken surrounding rock can bear the stress together. Shallow stress is transmitted to the deep stable surrounding rock mass, and the self-supporting capacity of the broken surrounding rock increases. To distinguish the mechanism of action of the anchor rod and anchor cable, the compression structure formed by the anchor rod is termed the shallow bearing ring, and that formed by the long anchor cable is termed the deep bearing ring. The interaction of the two load-bearing rings forms the load-bearing structure of the superimposed arch force transmission system^6^.

**Figure 8:**
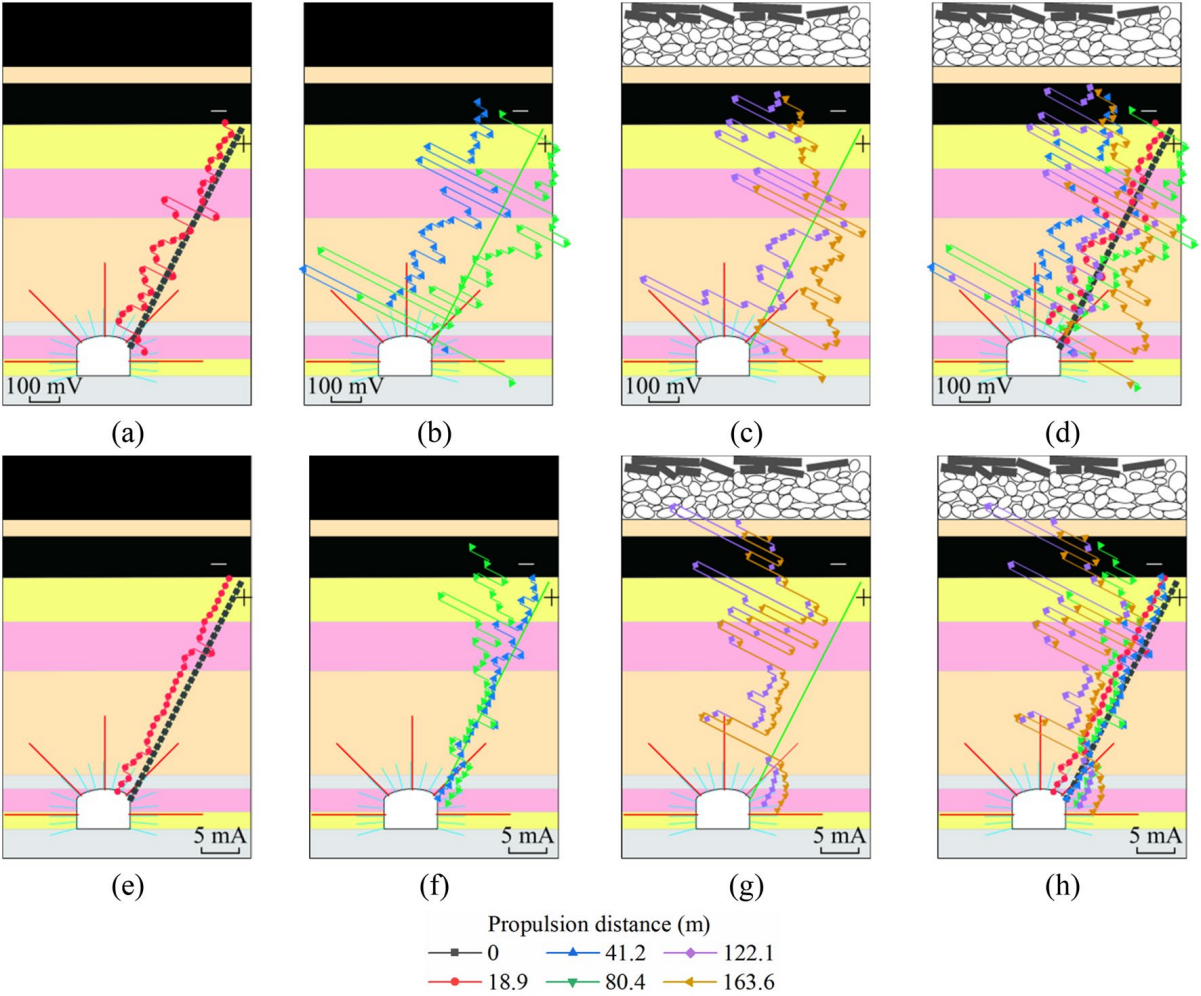
Relationship between the corrected data curve of geoelectric field and the surrounding rock formation of the roadway. (**a**)–(**d**) self-potential data; (**e**)–(**h**) excitation current data.

**Figure 9:**
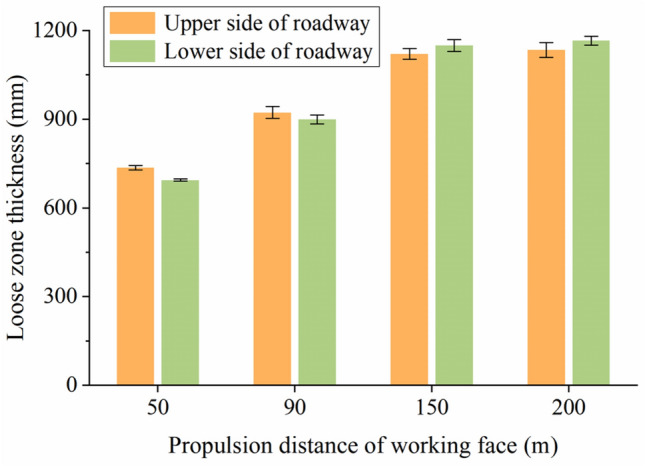
Change in thickness of the loose roadway-surrounding rock zone near the monitored section.

To more intuitively compare the variation laws and differences of various parameters in roadway surrounding rock under the influence of mining. We subtract the first set of background data values from each set of data values. The corresponding relationship between data curve and roadway surrounding rock layer after geoelectric field difference is shown in Fig. 8. During the mining of the working face, using LTD-2100 geological radar and gc400mhz antenna, the thickness change of roadway surrounding rock loose zone near the monitoring section was detected for many times. The detection targets include fine sandstone layer and sandy mudstone layer on the side wall of the roadway (Fig. 9). The effective test depth of on-site geological radar is within 2000 mm, and the thickness detection error is less than 6%. The boundary of loose zone of roadway surrounding rock is the boundary between broken structure rock mass and complete structure rock mass. The physical properties of rock mass on both sides of the interface are obviously different. The development thickness of the loose circle can be judged by the interface with obvious waveform change in the detected radar electromagnetic wave scanning results.

The potential factors that may affect the field test results mainly include the following aspects: (1) Metal support structure on roadway surface; (2) Contact between geological radar and roadway surrounding rock; (3) Selection of geological radar antenna frequency, etc. In order to minimize the impact of the above factors. In the actual test process, we try to reduce the geological radar directly passing through the metal structure and remove the metal equipment in the roadway. The geological radar shall be close to the surface of roadway surrounding rock for movement detection as much as possible. The selection of radar antenna takes into account the needs of detection depth and detection accuracy. At the same time, the geological radar test area is fixed every time, and the length of the survey line is the same. Taking the average thickness obtained by each detection as the final result, the test has continuity, contrast and reliability.

During the initial stage of mining, the rock mass in the monitored area is minimally affected by the mining stress, the geoelectric field data show no change (Fig. 8a,e), and the loose roadway-surrounding rock zone is approximately 700 mm. When the work face approaches the monitored area, microcracks in the floor rock near the coal seam of the working face form, expand, and permeate into one another. Macrofractures form in the rock mass in some areas. The excitation current of the floor crack development area significantly decreases, and crack development produces a large amount of free charge, which causes fluctuations in the natural potential. When superimposed with multiple stress fields, the range of the loose roadway-surrounding rock zone increases to approximately 910 mm. The excitation current in the shallow bearing zone does not drop due to the expansion of the loose roadway-surrounding rock zone and slightly increases. The excitation current in the deep bearing circle shows a downward trend (Fig. 8f), and the spontaneous potential fluctuates (Fig. 8b). Due to the impact of multiple stress fields, the loose circle in the shallow bearing circle expands. Because the electrode sensor in the cable is not coupled to the original rock and is placed in the grouted rock mass of the monitoring hole, when the tensile and compressive stress in the shallow bearing circle does not exceed the load limit of the grouted rock mass in the monitored hole, the grouted rock mass does not produce macroruptures. The increase in excitation current demonstrates that the grouted rock mass is anchored by the anchor rod to form a compression zone. During mining, due to regular periodic pressure on the working face, the excitation current in the shallow bearing circle shows multiple step-like increases, and due to the continuous change in the relative spatial relationship between working face and monitored area, it gradually decreases and stabilizes. During the mining process, to remain stable, the shallow bearing circle transfers a part of the stress to the deep bearing circle, whereas the grouted rock mass in the deep bearing circle is surrounded by mudstone and shows poor physical and mechanical strength. The mudstone layer in the bearing circle forms a stress concentration area and consequently macrofractures in the grouted rock mass, which decrease the excitation current.

As the working face gradually moves away from the monitored area, the geoelectric field response characteristics of different zones of the roadway-surrounding rock within the control range of the monitored hole are obvious (Fig. 8c,g). During the mining of the working face, the floor damage depth is approximately 14 m at the boundary between fine sandstone and mudstone. The structure of the mudstone layer outside the deep bearing circle does not significantly change, and no macrofractures are formed. The mudstone in the deep control range of the bearing circle forms a stress concentration zone and consequently few macrofractures, and the rock mass in the shallow bearing circle, where the impact of multiple stresses is observed, shows improved bearing characteristics due to the action of the anchor bolt prestress, which prevents tensile and shear failure in the fractured surrounding rock mass and limits the development range of the loose roadway-surrounding rock zone. When the mining distance of the working face reaches 150 m, the range of the roadway loose circle increases to 1,130 mm, and the growth rate decreases to 3.67 mm/m. By the time the working face reaches 200 m, the roadway loose circle has stabilized at 1150 mm, the growth rate decreases to 0.4 mm/m, and the stability of the shallow bearing circle is sound with no instability or failure of the roadway-surrounding rock (Fig. 9). Data from multiple ground-penetrating radars show that although the supporting structure of each part of the roadway-surrounding rock is similar, due to the difference in lithology of the rock and the uneven and asymmetrical distribution of stress in the surrounding rock, the development of fissures, deformation evolution, and failure vary in each area (Fig. 9).

## Mutual change relationship among multiple parameters

### Analysis of the fluctuation law of geoelectric field data at each measuring point

Through the above analysis, the rock fracture damage under the superposition of multiple stress fields is closely related to the self-potential, excitation current and other geoelectric field data. Therefore, the relationship between self-potential and excitation current can be explored by taking the rock fracture damage as a bridge. This helps improve the comprehensive processing and utilization ability of geoelectric field monitoring data and lay a test foundation for the next step of parallel analysis and joint inversion of geoelectric field multiparameters.

**Figure 10:**
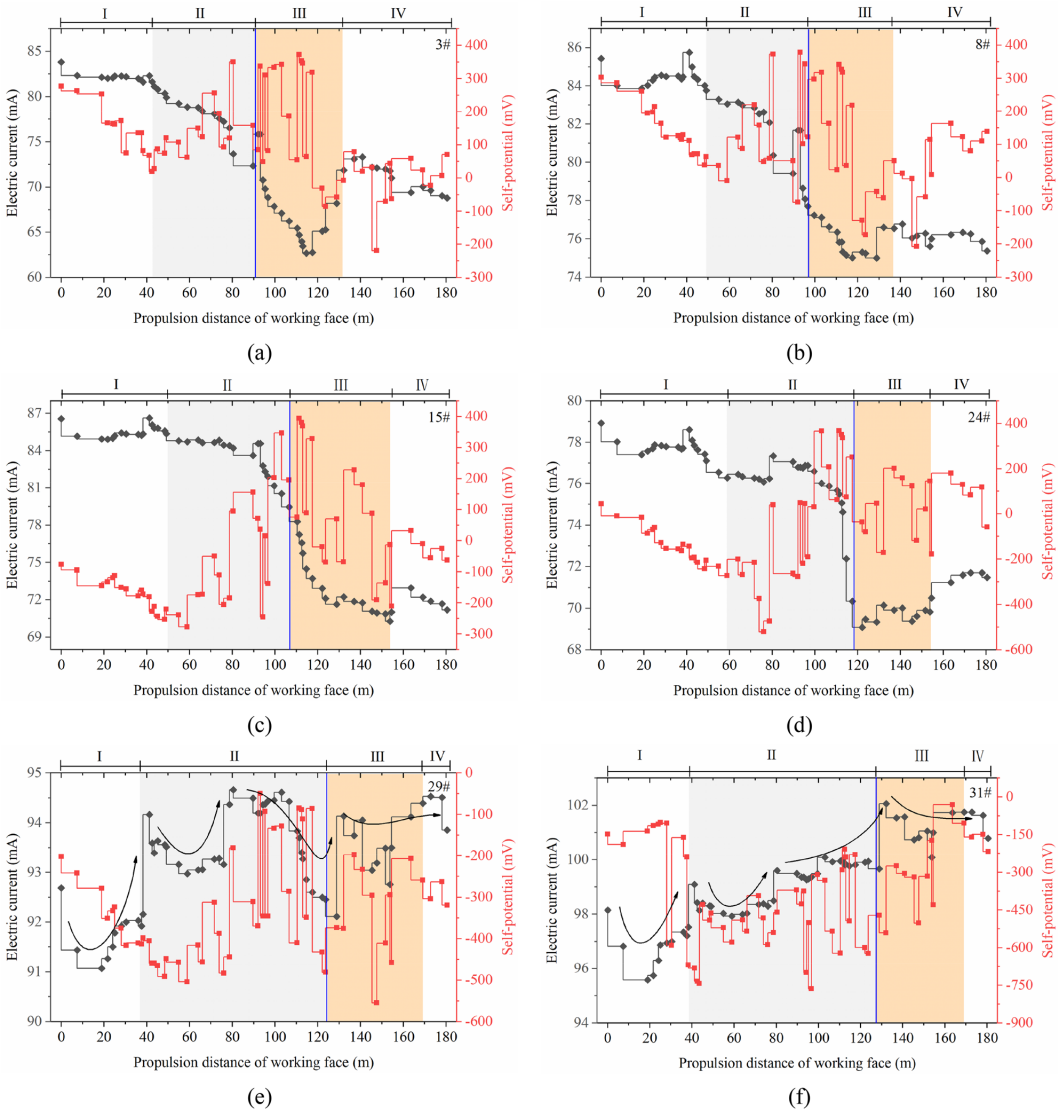
Relationship between geoelectric field data changes during mining.

Under the influence of the mining stress field, the rock mass fracture evolution law and characteristics of each measuring point in the floor damage area are basically consistent. In the early stage of mining, the rock mass in the monitoring area is less affected by the advance stress of mining, the primary fissures and new fissures in the rock mass breed and expand, the self-potential has a weak precursor fluctuation and slow decline process, and the decline range is generally within 200 mV; the excitation current data has no obvious change (Stage I). When the working face is approximately 48–58 m away from each measuring point, the monitoring area is strongly affected by the advance support stress, which makes microcracks in the rock mass continuously expand, mutually penetrate, and form macrocracks. The amplitude of the self-potential pulse, such as violent fluctuation, is more than 400 mV, and the excitation current has a significant downward trend with an average decrease of 6 mA (Stage II). When the working face is gradually away from the measuring point, unloading damage occurs in the floor rock mass of the goaf, and the fracture depth range and fracture degree significantly increase. When the working face is 20 m away from the measuring point, with overburden caving and compaction, further unloading damage of the floor rock mass is inhibited, and cracks in the broken rock mass exhibit extrusion or even closure. The amplitude of the self-potential fluctuation is obviously weakened, and the excitation current slightly increases. The change in rock mass in the shallow part of the floor damage area is more obvious (Stage III). When the working face is 40 m away from the measuring point, the distribution of the rock stress field in the floor damage area is basically stable (Stage IV) (Fig. 10a–c).

When the mining length of the working face reaches 60 m, the stress concentration of the mudstone layer in the anchor cable control area begins to appear, and fracture development is accelerated due to the superposition of multiple stress fields. The amplitude of the self-potential sharply fluctuates to 900 mV, and the excitation current continuously decreases (Stage II). When the working face is mined above the measuring point, the rock mass cracks propagate and form macrocracks. At this time, the excitation current sharply drops from 76 to 69 mA, and the self-potential fluctuation amplitude reaches 600 mV. Until the working face is 35 m away from the measuring point, the excitation current rises to 71 mA (Stage III) (Fig. 10d). However, when each measuring point in the floor damage area is 22 m, 25 m and 45 m away from the working face, the excitation current data are reduced to the lowest value, and the sharp fluctuation of self-potential is weakened. On one hand, the damage of the floor in the goaf after mining will be aggravated, and the risk of water inrush in the goaf increases. On the other hand, under the influence of the superposition of multiple stress fields, the fracture mechanism of the deep bearing circle of the roadway is different from that of the shallow damage area of the floor.

The bearing capacity of the shallow bearing circle of the roadway supported by the combined support of anchor rod and cables is obviously enhanced. Under the influence of dynamic pressure, the stress transmission distance and degree of the shallow bearing circle of the roadway are significantly greater than those of other strata. During the monitoring period, the excitation current presents a step-by-step overall rising process, but each time the current increases, there will be a small drop phenomenon. This is considered a dynamic self-adjusting process of the stress concentration and release of rock mass in the shallow bearing circle of roadway under the superposition of the mining stress field and support stress field. The existence of this process is an important sign of good structural stability of the surrounding rock in the shallow bearing circle of the roadway. During the period of severe mining, the accumulation and movement of electric charge in the floor damage area also affect the fluctuation of the self-potential in the shallow bearing circle of the roadway (Fig. 10e,f).

### Data movement trajectory and distribution at different stages

**Figure 11:**
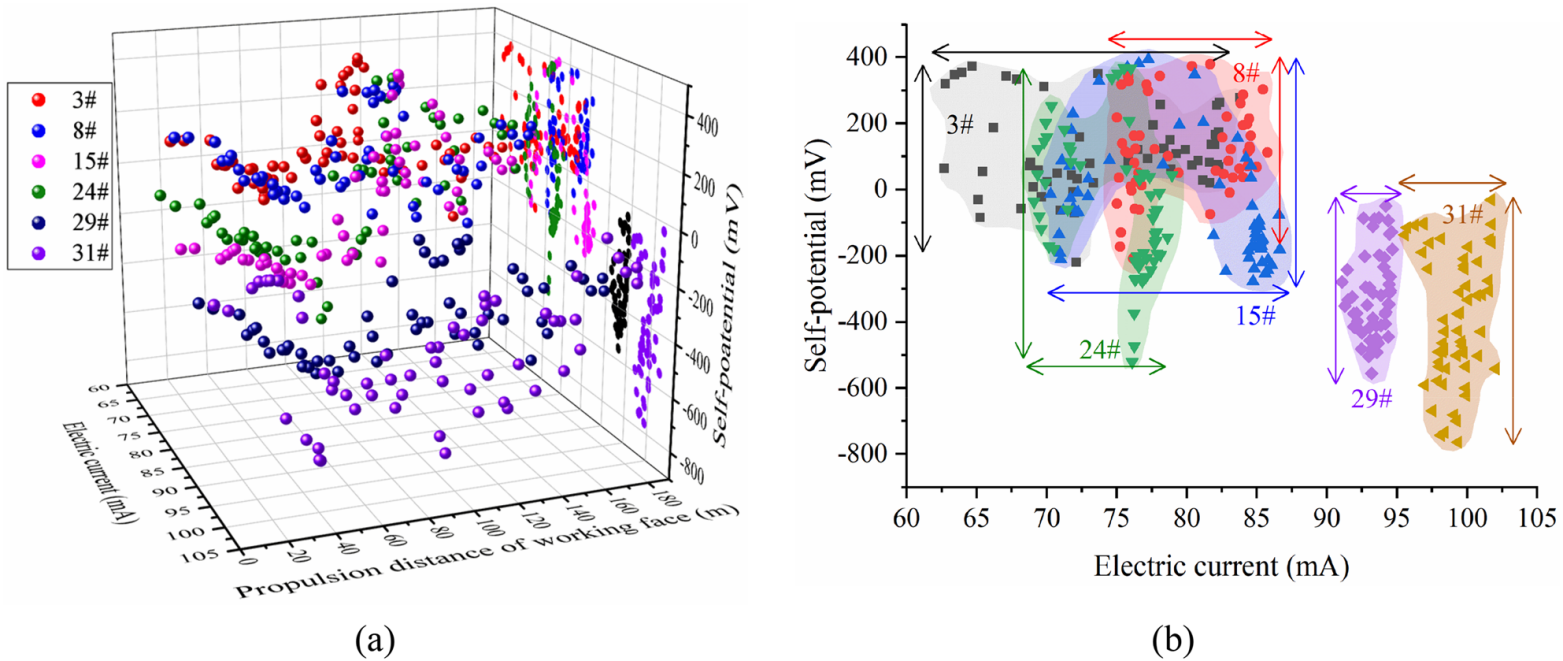
Scatter plot of geoelectric field data during mining. (**a**) 3D scatter plot. (**b**) 2D scatter plot.

Further analysis of the movement and distribution of the geoelectric field data at each measurement point in the mining process shows that there are certain regular spatial characteristics. In the early stage of mining impact (Stage I), the self-potential and excitation current data correlate; in the period of severe mining impact (Stage II, III), the data points sharply fluctuate in space; in the later period of stable mining (Stage IV), the changes in the two parameters tend to be stable (Fig. 11a). The geoelectric field data in rocks with cracks have a wide range of fluctuations, primarily on the upper-left side of the graph. The self-potential span is concentrated at – 300 to 400 mV, the excitation current span is concentrated at 65–85 mA, and the geoelectric field data in the shallow bearing ring of the roadway are distributed in strips on the lower-right side of the graph (Fig. 11b).

**Figure 12:**
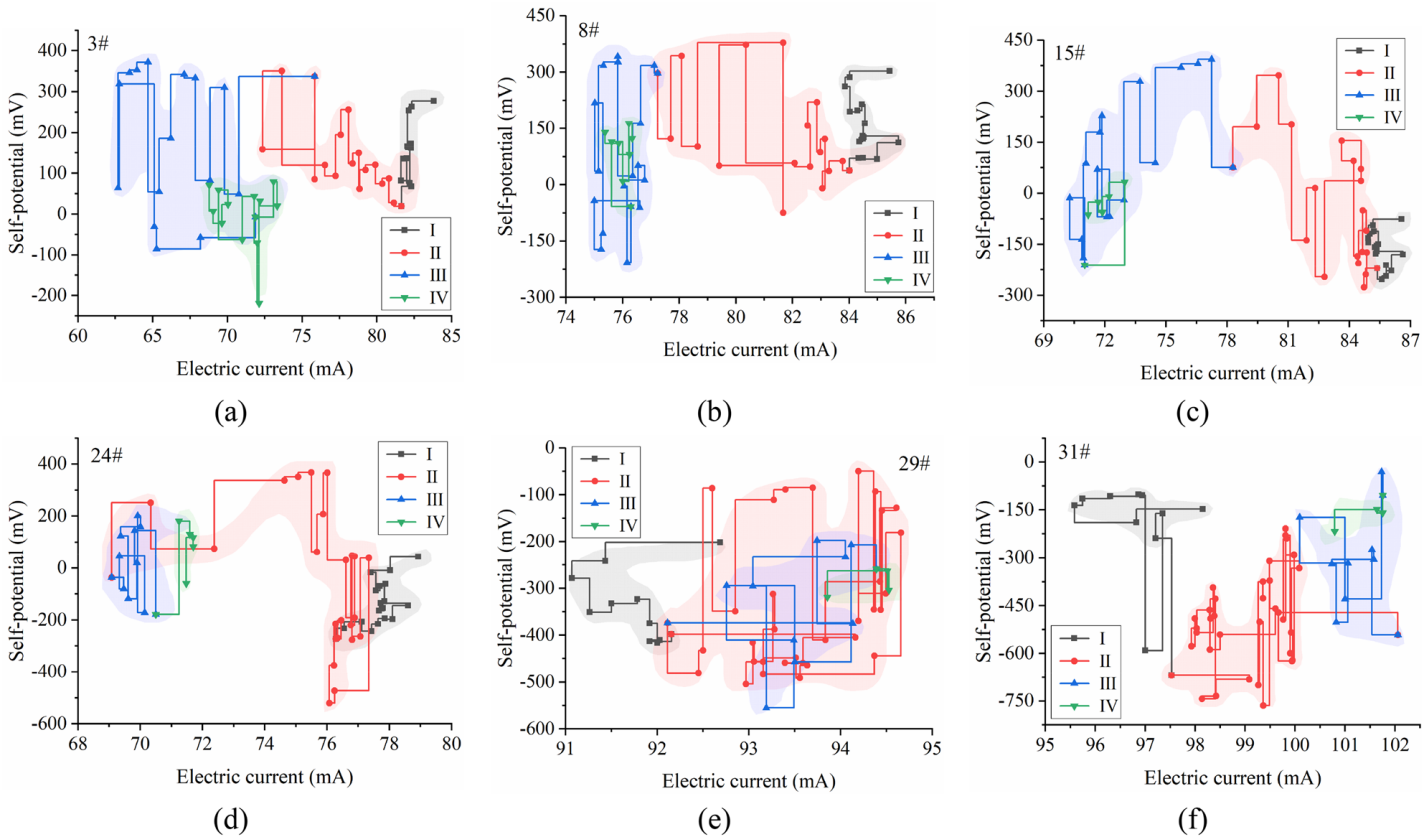
Distribution of the trajectory of the geoelectric field data at measurement points.

Analysis of the data trajectory of the measurement points at different stages affected by mining shows that measurement points #3, #8, and #15 have similar data trajectories, and the data trajectories in phases II and III are scattered in a larger area on the graph. The data trajectory distribution of each phase of measurement point #24 is more concentrated, and the trajectory of each measurement point is from right to left. The data of measurement points #29 and #31 slowly appear from left to right with a continuous back and forth fluctuation (Fig. 12). The trajectory and distribution range of the data of each measuring point at different stages are related to the development of cracks and damage in the rock mass at that location and the complexity of the rock mass subjected to the superposition of multiple stresses. The development of cracks and macrofractures in the shallow damage zone of the floor is directly related to the mining stress. Therefore, the trajectory of the measurement point data in this area is affected by mining. The shallow bearing circle and deep bearing circle of the roadway are simultaneously affected by the superposition of the mining stress and supporting stress, and the mudstone in the deep bearing circle shows a stress concentration and ruptures, whereas the shallow bearing circle does not show a continuous fracture process due to the stronger stress-bearing capacity.

## Discussion

### Accuracy of the test results

**Figure 13:**
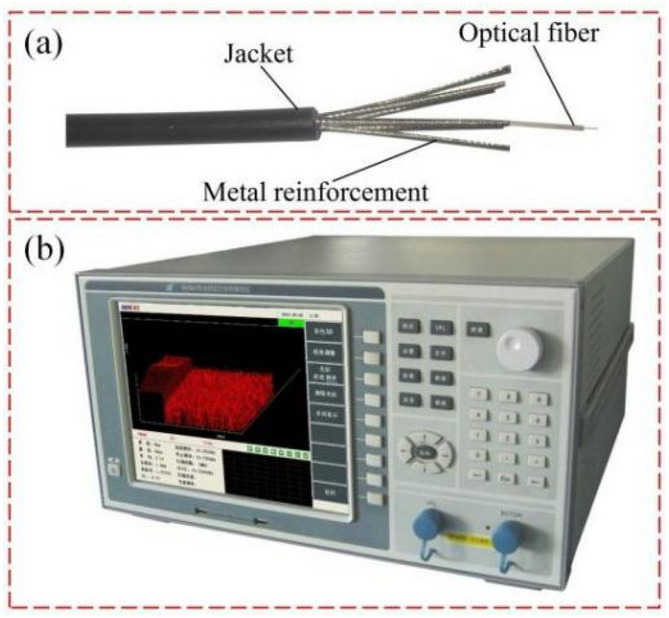
Strain field monitoring equipment. (**a**) Structure diagram of reinforced strain sensing optical cable; (**b**) BOTDR optical fiber demodulator.

In the monitoring borehole, we also carried out distributed strain optical fiber test, and obtained the strain data of rock mass damage during mining. The strain test equipment and optical cable are shown in Fig. 13. The existing literature has introduced in detail the performance parameters, field construction and test result analysis of optical cables and equipment^10,11,17,18^. Therefore, this part will not discuss the principle, test effect, influencing factors, judgment standards and other related issues of this technology. The following mainly focuses on the strain field test results to verify the reliability of the geoelectric field test results.

**Figure 14:**
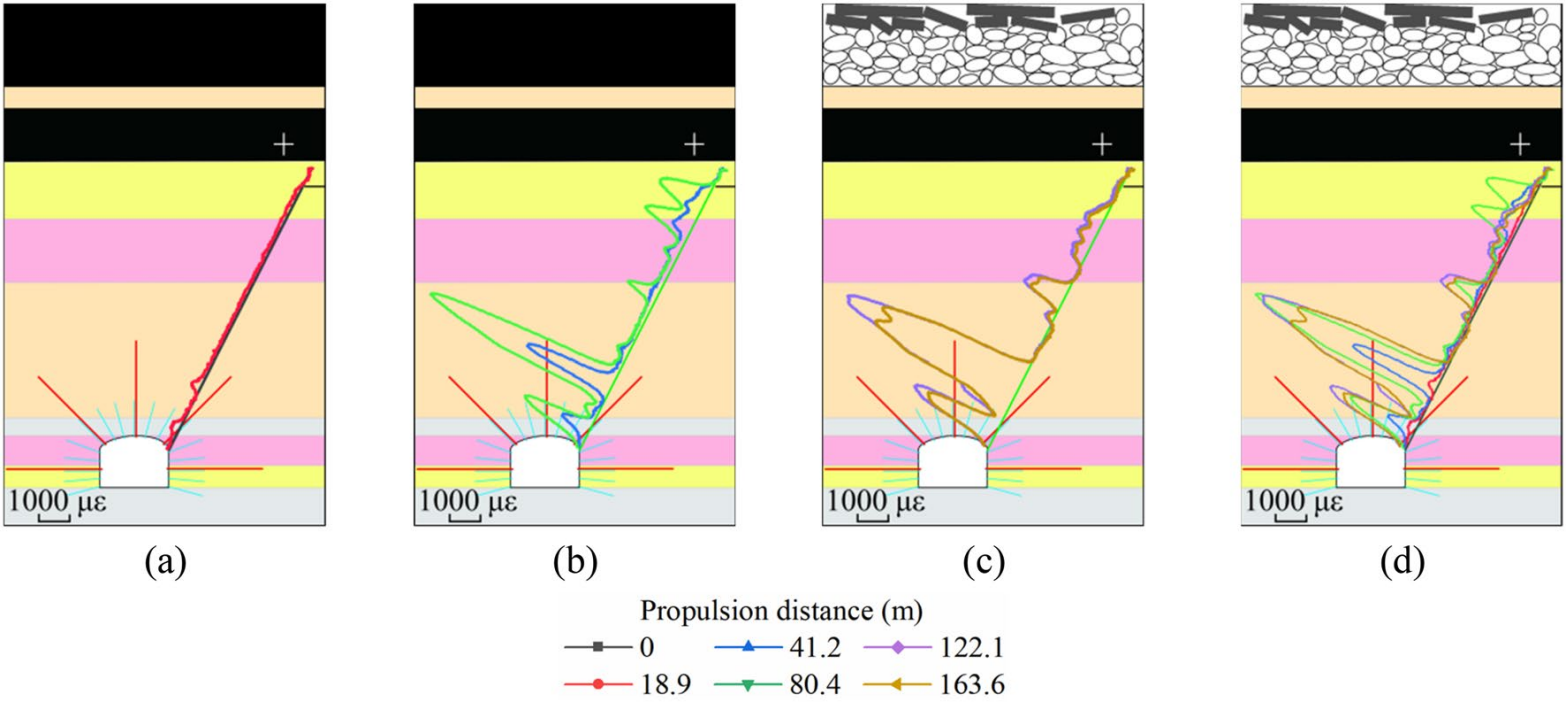
Correspondence between optical fiber strain data curve and surrounding strata of roadway.

The load strength of roadway surrounding rock mass under strong support such as bolt is significantly enhanced. The transmission distance of mining stress field in roadway surrounding rock is obviously farther than that in other strata. At the initial stage of mining, the stress field in the rock mass near the roadway changes first (Fig. 14a). With the mining approaching, the strain in the surrounding rock of the roadway increases significantly, and a stress concentration area is formed in the mudstone layer of the deep bearing circle. When the stress of mudstone exceeds its bearing strength, macro fracture occurs because the support structure can not effectively control and restrict the deformation and failure of rock mass in this area. In this process, the excitation current data decreases greatly. The shallow bearing ring of roadway is also affected by the superposition of multiple stress fields. Due to the effective control constraints of bolt and other supporting structures, although the strain value has changed greatly, it has not caused the structural damage of rock mass. During this period, the change of excitation current data is also not obvious. The floor rock near the working face is gradually affected by the advance bearing stress. The strain captured by the optical fiber in the rock stratum increases. Due to the lack of support in the rock mass in this area, there are damage cracks in the floor rock mass under the influence of mining. The strain field and geoelectric field data changed significantly (Fig. 14b). As the mining distance of the working face exceeds the monitoring area, the internal stress of the floor rock mass decreases and tends to be stable. In this process, the geoelectric field data is also in a relatively stable state. However, the stress field in roadway surrounding rock does not decrease significantly. It also shows that the surrounding rock mass of roadway controlled by bolts can bear a large range of stress field changes for a long time, and the surrounding rock structure of roadway remains in a relatively stable state (Fig. 14c,d).

By comparing the strain field obtained by field monitoring with the geoelectric field data, it can be seen that the test results are basically consistent. During this period, the real-time change law and characteristics of multi physical field data can be verified each other, and it is also consistent with the results in the existing research literature. In complex geological environment, geoelectric field testing technology is also reliable and practical. The accuracy and precision of measured data results can meet the interpretation and analysis of various research purposes and processes at this stage.

### Geoelectric field response of rock mass damage during mining

In real time, accurately grasping the response characteristics of the geoelectric field of internal fracture expansion is of great engineering significance for the scientific guidance of the stability control of deep underground rock mass engineering and promoting the safe mining of deep mine resources.

During the working face mining period, the change trend of self-potential of each measuring point in the monitoring area is relatively consistent; in particular, the characteristics of pulse-like violent fluctuation in the stage of severe mining influence are more obvious. This is due to the acceleration of crack propagation caused by stress mutation, which leads to charge accumulation and instantaneous emission and subsequently produces continuous charging and discharging phenomena. The generated electric charge will rapidly move, gather at the surrounding rock (including the unbroken rock) and affect further rock, which causes obvious abnormal changes in the natural electric field in the monitoring area. This phenomenon affects the rock mass in a large range of the roof and floor of the mining face. The amplitude of the self-potential fluctuation is relatively small only in the rock without damage^23^. However, the excitation current is in response to the change in macroscopic conductivity of the surrounding rock mass. In the early stage of mining, the development of microcracks in the rock mass is limited, and the conductivity of the rock mass changes only when the damage and failure of the rock mass reach a certain extent^25^.

When the working face was 50 m away from the monitoring area, we performed continuous geoelectric field monitoring. According to the captured self-potential signal, when the mining floor rock mass gradually stabilized, the negative charge in the damaged rock body relatively gathered, while the positive charge showed the phenomenon of diffusion and accumulation into the mudstone layer, which continued until the end of the field monitoring project. Finally, compared with the initial monitoring, the self-potential in the fractured rock decreased by 250 mV, while the self-potential in the mudstone increased by 140 mV (Fig. 4a,d). The research results are consistent with Wu's physical model experiment, which shows that the fracture development degree of rock masses in different areas and the change in self-potential have obvious changes. A higher degree of fracture development corresponds to a greater decrease in self-potential. For rock without damage, the self-potential increases and can be stable for a long time^23^. According to this study, we also obtain a new understanding: in deep underground space, the electrons released to the surrounding environment of the damaged rock mass in the stage of severe mining impact will not return to the damaged area in a short period of time. The positive charge takes the stable rock stratum as the unit, gradually accumulates in the undamaged rock mass, and will be stable for a long time.

As a type of geophysical method of electrical exploration, geoelectric field signals can reveal the evolution process of rock damage and failure at a deep level. The self-potential focuses on capturing the microelectrochemical field changes of rock masses, such as microcrack incubation, charge accumulation and migration, and chemical action. The excitation current mainly reflects the evolution of the rock structure physical field, such as the fracture development, rock fragmentation, and rock stress concentration. In addition, the self-potential has higher response sensitivity to the development and expansion of microcracks and the precursor information of fractures than the excitation current. In the aspect of advanced prediction of dynamic disasters related to rock damage, self-potential has obvious advantages in the time domain. However, the excitation current has obvious advantages in describing the evolution process of macrocracks in different regions, especially in the accurate determination of the spatial differential failure of rock masses. In the complex stress field environment, the failure of the spatial difference of the rock mass can also be accurately identified by the excitation current data. These two electrical parameters can complement each other in the expression of monitoring results. Compared with the single-parameter test method, such as resistivity, the geoelectric field monitoring results can more comprehensively and finely describe the rock failure process. This paper provides guidance for further comprehensive use of geoelectric field testing technology to carry out accurate identification and early warning of rock mass damage and failure state in complex environment, and lays a foundation for parallel analysis and joint inversion of multi parameters.

## Conclusions

To study the mechanism of the time–space evolution of rock damage and fracture under complex conditions, this paper proposes to perform fine real-time monitoring research of the entire process using the geoelectric field monitoring technology. The research shows that the temporal-spatial response characteristics of geoelectric field signals are closely related to the stress distribution and damage evolution of the surrounding rock. Self-potential and excitation current test parameters complement each other in the expression of monitoring results. The two electrical parameters can fully and accurately describe the entire process of stress damage and fracture of rock masses in different regions under complex stress environments.

Some deficiencies remain in this study. For example: (1) Only one dip monitoring borehole is constructed in the field monitoring area, and the results cannot fully reflect the spatial damage evolution state of the surrounding rock in the roadway section. Multi-angle monitoring boreholes should be added in future field test work to monitor the full section roadway surrounding rock structure. (2) The test results can only qualitatively analyse the damage process of the rock mass but cannot quantitatively grasp the accurate damage degree of the rock mass in each period. It is recommended to construct verification holes near the monitoring area to conduct drilling TV or other test verification work and visually observe the real-time damage conditions such as crack development in the hole. (3) Some typical rocks can be selected to perform relevant indoor loading experiments to obtain quantitative characterization information of the geoelectric field in each stage of rock damage during loading. By improving the geoelectric field test in the complex environment of the entire space, we expect to gain a deeper understanding of the accurate identification of rock mass damage and fracture risk.
